# Collectanea

**Published:** 1832-08

**Authors:** 


### COLLECTANEA.

**Floriferis ut apes in saltibus omnia libant,**
**Omnia nos, itidem, depascimur aurea dicta.**

### PATHOLOGY.

*Case of Chronic Hydrocephalus cured by Puncture.* By Professor GRAEFE, of Berlin.

A child, whose head had from its birth been preternaturally large, but who was otherwise healthy, was, at four months old, admitted into the University Hospital at Berlin: it was then pale without being emaciated, and well made. The head, however, shewed symptoms of chronic hydrocephalus; the face was small in comparison to the cranium; the hair was fine, light-coloured, and very thin; the fontanels were widely open, and the sutures unclosed; the bones of the skull mobile, thin, and little advanced in their ossification: the greatest circumference of the head was eighteen inches and a quarter: fluctuation could be perceived every where, and especially at the anterior and posterior fontanels; when pressure upon one of which was made, the other presented a hard translucent tumor.

Not any of the remedial means employed had the least salutary effect, and hence M. Graefe determined to try whether puncture would afford relief.

Having compressed the great fontanel so as to determine the fluid towards the small one, he introduced a moderately-sized cataract needle, at first vertically into the fontanel close to the side of the bone, and then, giving it an oblique direction, carried it onwards about a third of an inch. The liquid, which was viscid, dropped out but slowly; the operator, therefore, withdrew the cataract needle, and introduced in the same way a fine trocar, and, as soon as the canula was opened, a transparent yellowish brown fluid gushed out in a free stream. In about half a minute the canula was closed, with the intention of subsequently reopening it after the lapse of a few minutes, which was done several times, the skull being, during the whole period, gently compressed by the hands of an assistant applied on either side. When twelve drachms of the fluid were discharged, the infant's eyes became suddenly dull, the pupil contracted, the countenance pale and altered, and the action of the heart and the pulse more feeble. The canula then was immediately withdrawn, the wound closed, and the head compressed by the application of straps of adhesive plaster.

These symptoms did not disappear for several hours, notwithstanding the exhibition of stimulating medicines, which were prescribed; and the child remained restless, slept little for the two following nights, cried much, and took the breast but seldom.

The same symptoms occurred after each subsequent operation, but it was found that the child became completely restored in from about ten to fourteen days. At first only about twelve drachms were evacuated after each puncture, subsequently twenty were discharged. Between the earlier times of operating, the little patient took, morning and evening, the eighth of a grain of calomel, and the sixteenth of a grain of foxglove; but this powder causing nausea, it was changed for two or three grains of calomel with magnesia, to be taken twice a day, two or three times a week, the head being bathed assiduously with squill vinegar and water, just warm; for after cold applications, which were tried several times, the infant was always uneasy, pale, and faint, insomuch that convulsions were feared. The head diminished in diameter two or even three lines after each operation, and by degrees the dimensions of the skull were reduced to a conformity with the face and the rest of the body. The fluctuation, and the mobility of the cranial bones diminished; the sutures closed, and the general state of the patient was improved. The punctures were repeated eleven times at the following periods during the year 1829: viz. the 8th, 15th, and 23d of January; 19th of February; 5th and 19th of March; 19th and 27th of April; 5th and 17th of May; and 23d of June. The liquid evacuated became thicker and more coagulated towards the end. After the last operation on the 23d June, no further fluctuation was perceived; the little fontanel and all the sutures were closed, the great fontanel alone remaining slightly open. The child grew, and even after the third operation it had already a better appearance, and after the ninth it began to articulate certain words, and also to walk: at ten months old it ran alone, and spoke as well as children of that age usually do. At the end of June, its head measured in the greatest circumference eighteen inches and three quarters.

On the 26th November, 1830, the child being then two years and a half old, was alive and well, and was presented to the Society of Medicine at Berlin. —*Graefe und Walter's Journal der Chirurgie.*

---

*Case of Propagation of Ringworm by Contagion.* M. Collineau communicated to the Académie Royale de Médecine the following fact. In an establishment which contains between eleven and twelve hundred females, there is a particular department appropriated for the reception of girls from ten to sixteen years of age, in which they have communications only with each other, and with the persons intrusted with the care of them. In the month of August, 1831, a child with a ringworm on her shoulder, about ten or twelve lines in diameter, was admitted into this establishment. Two months afterwards, one of her companions had a similar ringworm on her arm, and also on her left cheek. At the end of four months, the greater part of the others were affected with the same disease, attacking the arms, thighs, neck, hands, &c. By the 7th February, no more than three out of seventeen remained who were exempt from the disease, and of these one subsequently, as well as the matron, was affected.

It is impossible, observes M. Collineau, to doubt that these ringworms were communicated by the child admitted into the establishment in the month of August, as previously there were no affections of that kind in the house, and as the dormitory in which she slept was the only one affected. The situation is healthy, and the diet good. The author in reporting these cases of contagion, does not the less acknowledge the rarity of the communication of scaly cutaneous diseases.—*Archives Générales.*

---

*Observations on the spontaneous Amputation of the Limbs of the Fœtus in Utero, with an Attempt to explain the occasional Cause of its Production.* By W. F. Montgomery, m.d., m.r.i.a., Professor of Midwifery to the King and Queen's College of Physicians in Ireland.

Several of the writers who have expressly treated of the pathology of the fœtus, in their enumeration of the accidents to which it is liable during its uterine existence, mention the fact of the occasional separation of portions of the limbs; and especially Desormeaux, in his elaborate and able article on the "œuf humain" speaks of it as an instance of spontaneous amputation, which he attributes to the effect of inflammation and gangrene.

But none of these authors appear to have witnessed or examined any case of the kind themselves, nor can I find a satisfactory reference to any such case in any author within my reach, except those which are now to be noticed.

In the London Medical and Physical Journal, vol. liv. p. 38, Mr. Watkinson relates the following case. Being called to a lady, aged twenty, in her first labour, which was natural and easy; when the child was expelled, "he discovered that the left foot had been amputated a little above the ankle, and the part nearly, but not quite healed."

The child was alive, and gasped for twenty minutes, when it expired. The mother had only gone seven months. On examination after the birth, the foot was found in the vagina, and it also was nearly healed. There did not appear to have been any hemorrhage from the limb; the separated foot was much smaller than the other, which was rather turned inward; it shewed no mark of putrefaction, but appeared to be in a state of perfect preservation. The mother said she had not been frightened, nor had any unpleasant circumstances occurred in her family to give her the least uneasiness; her circumstances were sufficiently easy to render unnecessary any over-exertion on her part.

The parts here described were examined by the editors of the Journal, and a sketch of them is subjoined to the description.

M. Chaussier examined two cases in which separation of a part of the forearm had taken place before birth; and in a third case of the same kind, he found the separated portion of the arm and the hand lying in the membranes, and, as in Mr. Watkinson's case, the stump was healed.

Chaussier also attributes the accident to gangrene, as the cause which would most obviously account for its production; but it does not appear from his account, that there were present any of the pathological evidences of that condition; and indeed, as a general explanation, this appears hardly admissible when we recollect that, in the first case related, the child was born alive, and neither the stump nor the part amputated shewed any symptom of disorganization or disease, nor was the latter even discoloured.

Without pretending to discuss the different causes likely or unlikely to produce so remarkable a change, I shall proceed at once to describe the case which occurred to myself, and which appears to me to offer one explanation at least of the phenomenon under consideration.

About three years since, I was suddenly called on to see a patient who was miscarrying in the fifth month, with violent hemorrhage. On examination, I found the fœtus partly expelled from the uterus, and lying in the vagina, from whence I readily removed it by slight pressure with my finger; the cord was broken off, at about an inch and a half from the umbilicus; the secundines were retained for three or four days, without any return of hemorrhage, and were then expelled whilst the patient was evacuating the bowels. She recovered well.

Observing the unusual figure of the head, I laid by the fœtus for examination; and having placed it in clean water for the purpose, I was greatly struck with the appearances it presented.

The shape of the head was altogether deformed and monstrous, and the brain, which at the left side was covered only by the common integument, towered upwards like a helmet over the head; but the circumstance which most forcibly attracted my attention was the appearance of distinct ligaments on the limbs; and examining closely, I found them in the following condition:

There were distinct threads of, I presume, organized lymph passing from both hands to the legs; at one end these threads, which very much resembled the kind of thread called ardis, had formed a complete ligature round the middle of each hand, causing a distinct depression where it passed, the part of the hand below it being almost completely undeveloped; from the hands these threads descended, at both sides towards the legs, which were crossed, and surrounding them in this position, just above the ankles, so tightly, that fully two thirds of their whole thickness were thereby divided, without, however, any breach of the skin having taken place, nor was there the slightest appearance of disorganization or discoloration of any of the parts; but, as were the hands, the feet also were imperfectly formed, totally undeveloped, and of course misshapen. These circumstances are very accurately represented in the drawing executed by my zealous and accomplished pupil, Mr. J. Bullar, and the fœtus itself is preserved among the preparations in my museum. The mother was about twenty-five years of age, and at the time labouring under fever, but had been previously in perfectly good health, and had not met with any accident or circumstances of either bodily injury or mental agitation.

From the condition of the limbs thus produced, and the impossibility of the parts below the ligatures continuing their growth under such circumstances, it seems exceedingly probable that had the child continued to live and grow, the parts of the legs below the ligatures would have been separated, and thus undergone spontaneous amputation. The formation of these threads, and particularly their application so as to stricture the limbs, are circumstances in explanation of which I do not feel prepared even to hazard a conjecture, nor can I find in the authors who have treated of such subjects more than a mere allusion in some of them to the fact, that the fœtal limbs are occasionally separated, and by all it seems taken for granted that mortification is the agent of the change.

The only passage which I can find apparently relating to the state of the parts just now described, is in the Elementa Physiologiæ of Haller, t. 8, p. 135; it is as follows: "Huc faciunt alius fetus, cui artus retracti, compressi, ligamenta stricta, &c.," the last phenomenon he quotes from one of Rœderer's works, which I regret I cannot find in any of our libraries, and am therefore unable to ascertain how far it might bear upon or illustrate the fact before us. It does not appear from any passage in his writings, that Haller was himself aware of any such case from personal observation. He gives, indeed, a long list of extraordinary mutilations in the fœtus, attributed by authors to the effects of mental emotions in the mother, or of accidents sustained by her; but he immediately pronounces most of them to be "adeo fabulosa ut fidem auferant," and he obviously considers such cases as the result of imperfect development or of malformation, and not of separation or removal of the parts already formed, objecting to the authors who have furnished such descriptions, that they cannot even quote one instance in which "manus truncata, aliusve artus in membranis fetus seorsim a corpore repertus sit;" two such instances at least have, in the foregoing remarks, been laid before the reader, and with regard to the case which I have added, I will only venture to say, that it appears to me to afford at least one solution of so mysterious an occurrence; and should it appear, as of course it must, that the explanation thus supplied is so far unsatisfactory, that it is itself the result of a process equally inexplicable, it should be recollected, that very many indeed, if not all the physiological and pathological results which we witness, are, as to the mode of their production, enveloped in a similar cloud of obscurity: I think the fact which I have here added, leads us at least one link farther in the chain of causes and effects, and if so, even though the advance should be but of one step on the road to knowledge,

"Est quôdam prodire tenus si non datur ultra."

*Dublin Journal of Med. and Chemical Science.*

---

### MEDICAL JURISPRUDENCE.

*Medico-Legal Examination of two Cases of Death from Injuries of the Head, involving important Questions in Medical Jurisprudence.* By Alexander Watson, Esq. F.R.C.S.E.

Case I. On January 20th, 1827, I was requested by the sheriff of the county of Mid-Lothian to accompany Mr. Liston and Mr. Mackenzie at the inspection of the body of Alexander Clark, and report along with them as to the cause of his death.

Clark was a carter, about twenty-five years of age. Shortly after five o'clock on the morning of the 8th of January he had gone along with another man to an infamous house, kept by a man named Mackenzie, to get a dram. His companion left him, and was succeeded by two women of loose character, who picked his pocket of a purse containing about fifteen shillings. Clark snatched the purse from one of the girls; she immediately called out that she had been robbed, and Mackenzie came to her assistance. A scuffle ensued. Mackenzie fought furiously with Clark, threw him down, kicked him severely in several places, cut his nose with a blow, and turned him out of his house. In this scuffle Mackenzie tore the purse forcibly from Clark, emptied it of its contents, and put it into Clark's pocket as he was leaving the house.

Clark went immediately home and told his friends that he had been "robbed and murdered." He was confined to bed with the bruises he had received; but the medical gentleman who then saw him did not consider his life in danger.

On the 13th, he was able to go out for a short time upon business. But on the 14th, he complained to his friends that he felt contraction of the mouth, stiffness of the jaws, difficulty of swallowing, and dimness of the eyes. He could not take food on account of the contraction of his mouth and difficulty of swallowing.

had

On the 15th, necessity made him go out to his work; but he felt himself so unwell that he was obliged soon to return home. He was able to take a little spoon-meat, but, being unable to open his jaws, he could only get the spoon a short way into his mouth. He was obliged to go out again in the evening for half an hour to attend to a horse that was under his charge. When out he felt very unwell, and complained that he felt as if he would fall down. On his return home he went to bed.

On the 16th, his mouth was almost quite closed. Medicines could only be got into his mouth where a tooth was wanting. He was able to identify Mackenzie as the person who had injured him, and to make a declaration in presence of the sheriff. He continued to become worse till the 19th, when he died, having been previously seen by Mr. Liston and Mr. Mackenzie. On the day subsequent to his death we made a careful inspection of his body.

Externally there was a small ragged lacerated wound upon the nose, at the lower extremity of the suture which unites the two nasal bones. There were also marks of contusion upon the right elbow and left hip-joints.

On dissection there were found several small portions of extravasated blood under the integuments of the head, particularly above the right eye. The brain was natural; the vessels filled with blood; some serum in the ventricles and the spinal sheath. The posterior part of fauces was of a dark-red colour, from congestion of the vessels of the lining membrane. This appearance was distinctly circumscribed, and terminated at the upper end of the œsophagus, root of the tongue, and posterior part of the nostrils. The membrane lining the air-passages had a similar appearance, and contained a considerable quantity of fluid tinged with blood and purulent matter. The larynx was open and dilated; there was some congestion of lungs; but the abdomen was natural.

We reported to the sheriff that Clark had died from "locked jaw," (*tetanus;*) and that the appearances on dissection were not of themselves sufficient to account for his death.

An important question then occurred, whether the tetanus of which Clark died had been caused by the injuries he had received, or imprudent exposure to cold while suffering from these injuries, or partly from both of these causes? It will be observed that he received the injuries on the 8th of January. He did not go out till the 13th, while on the 11th he had complained to his friends of the tetanic symptoms. There was, therefore, no exposure to cold, or any other cause which could have occasioned tetanus, between the receipt of the injury and the time at which its symptoms first appeared. Though tetanus sometimes occurs from injuries in this country, it is by no means common; and it very rarely takes place from exposure to cold. We therefore stated as our opinions at the trial of Mackenzie, which took place on the 14th of March, 1827, that Clark's death had been occasioned by tetanus, resulting from the injuries he had received on the 8th of January. The charge of assault was clearly proved against Mackenzie, who had inflicted the injuries. But the charge of murder was departed from by the public prosecutor, with the approbation of the judges; because the injuries Clark had received were not of a mortal nature, and had been inflicted without any intention of committing murder; and likewise because locked-jaw was not a necessary or usual consequence of such injuries. The jury, therefore, returned a verdict of culpable homicide, and Mackenzie was sentenced to transportation for fourteen years.

Case II. On the 16th of July, 1831, Dr. Christison and I proceeded, by warrant of the sheriff, to inspect the body of Mrs. Stevenson or M'Cormick, in order to report as to the cause of her death.

This woman had lived very unhappily with her husband and a daughter of his by a previous marriage. They were all much addicted to intemperance, and were people of degraded character. M'Cormick had served in the army, for which he received a pension. But unfortunately, when this was paid to him, he could not refrain from drinking to intoxication, so long as it lasted. His conduct at such times was very outrageous. In the brutal conduct of beating, kicking, and dragging his wife about by the hair of the head, his daughter joined him; and on the day previous to the death of his wife he had been seen by several of his neighbours to beat and maltreat her. Several persons saw him give her severe blows with his fist on the head and face. In the evening she took refuge in the house of a neighbour, to whom she complained much of her head, and declared "they had murdered" her. Next morning she was seized with convulsions and coma, of which she died in a few hours. At the time she died, a student of medicine, who had been called to see her, several neighbours, together with her husband and his daughter, were present. In presence of these bystanders the husband and his daughter accused each other of having murdered the deceased, at the same time swearing at each other the most horrid oaths, and using the most shocking epithets.

On inspecting her body, we found several marks of recent contusions on her face, head, and arm. The skull was not fractured, and was throughout three-eighths of an inch in thickness, being about three times the thickness of ordinary skulls. A slight bloody effusion covered the upper part of the right hemisphere of the brain, beneath the dura mater. At the anterior part of the middle lobe of the brain on the right side, there was a pretty thick coagulum of blood, which, when removed, appeared to amount in quantity to about half an ounce. The substance of the brain was firm and vascular; the lungs and heart natural, the latter coated with fat, and contained no blood; the arteries were natural; the kidneys small, and much diseased, having a mottled appearance, and changed in structure; the urine presented coagulated flakes on boiling; and the ovaria were enlarged, and converted into cysts filled with watery fluid.

Our report upon this case stated, that we had found several marks of contusion on the face, head, and other parts of the body, and an effusion of blood upon the brain sufficient to have occasioned her death; but that it was impossible for us to say whether this effusion of blood had arisen from violence or from natural disease. We also mentioned our having observed other parts of the body in a state of disease, but not of a nature to have caused sudden death.

At the trial of M'Cormick and his daughter for the murder of his wife, the case rested entirely on the medical evidence. Habitual maltreatment, and blows on the day before her death, was clearly proved against them. But the question came to be, whether the effusion of blood which had caused her death had been produced by violence, or had arisen from natural disease and intoxication? Several circumstances strongly concurred to favor the conclusion of violence as the cause. These were,

1. The infliction of severe blows on the head just before the fatal symptoms came on, sufficient to have occasioned the effusion of blood on the surface of the brain.

2. External marks of violence on the head.

3. Effusion of blood from natural disease rarely takes place on the surface of the brain; and seldom at all without either disease of the brain or of its arteries.

4. The symptoms and appearances were precisely such as might have been expected from such blows as the woman had received.

In giving our evidence, therefore, we concurred in opinion, that the fatal effusion of blood on the brain was more probably owing to the violence she had received than natural disease.

We gave this opinion as medical jurists, called upon to aid impartiality in the elucidation of the case. In such a case it could not have been more decided, but more especially in this one, on account of the drunken and irregular habits of the deceased: and the public prosecutor, with that laudable humanity and moderation which always characterize the administration of the laws of our country, departed from the charge of murder, on account of our professional opinion being uncertain. But, when taken along with the general evidence, which was very strong against the prisoners, the propriety of the decision come to may fairly be questioned; for there were several direct proofs of violence, and none of natural disease, having occasioned death; the conclusion seemed almost irresistible: but this was for others to decide. We performed our duty by stating the facts, our opinion, and the doubt which attended the case. The jury, however, had no alternative; they found both panels guilty of aggravated assault, and they were accordingly sentenced to transportation for fourteen years.

The general history of both the above cases is taken from the evidence adduced at the trials before the High Court of Justiciary.—*Edinburgh Med. and Surg. Journal.*

---

### MIDWIFERY.

*Observations on the Obstetric Operation of Turning.* By THOMAS RADFORD, Esq. Senior Surgeon to the Manchester Lying-in Hospital and Dispensary for the Diseases of Women and Children.

THE operation of turning is not limited in its application to one case, but is essentially demanded, and affords the only safe means of rescuing the female as well as her offspring from impending danger in a number of accidents occurring during birth. One method, if duly established as the best, will answer in all cases to which this operation is applicable. To shew the predicament in which the practice now stands, I shall quote the directions given by the writers most generally and deservedly esteemed in this country, as well as in France and America.

The rules established by Mr. Denman may be understood from the following passages:

"If both the feet should be lying together, we must grasp them in our hand; but if they be at a distance from each other, we may commonly deliver with one foot without much additional difficulty; though, as in some particular positions we cannot always turn the child, if it be large, by one foot, it is better to make it a general rule to bring down both feet together, when they are in our power." Dr. DENMAN's Introduction to the Practice of Midwifery, vol. ii. p. 235.

"The hand is then to be immediately carried into the uterus, and, if we have decided on turning, upwards until the feet be found. Both feet are to be grasped between our fingers, and brought down into the vagina, taking care that the toes be turned to the back of the mother."—BURNS' Principles of Midwifery, &c. p. 316.

"Having passed the hand beyond the cervix, we carry it on bewixt the body of the child and the surface of the uterus, which is felt hard and smooth, from the tonic or permanent action of the fibres, until we reach the feet, both of which, if possible, we seize and bring down; but if we cannot easily find both, one is to be brought down into the vagina, and retained there. The child will be born with the other folded on the belly."—Idem, p. 367.

"In every turning case you are to introduce your hand by the side of the presenting part; as the feet are brought down this goes up. If both feet cannot be readily grasped and brought down together, bring down one first and then the other."—A Practical Compendium of Midwifery, &c. by the late Dr. GOOCH, p. 236.

"The established practice," says Dr. Merriman, "then, is for the operator to pass his hand into the uterus, to take hold of the feet, and bring them without the os externum; thus converting the presentation of the arm into a presentation of the feet."—Synopsis, p. 80.

Dr. Blundell, after recommending the accoucheur to grasp the feet in any mode he can, proceeds to say,

"If both legs are seized, the child will turn more easily. If you can grasp one leg only, let this be brought down. Often you may turn by one leg; but should it be necessary to draw down the other, the access to the second will be facilitated by the descent of the first. Should the seizure of the leg be impracticable, I would recommend you to lay hold of the knees, gradually working your fingers towards the feet, in the way here demonstrated."—Lancet, vol. i. p. 551.

The directions of the foreign accoucheurs are much of the same character.

" We hook the feet with the ends of the fingers a little bent, and bring them to the entrance of the vagina, making them pass over the breast and face of the child. When we can take hold of but one foot at once, we must take that which belongs to the side that the hand has passed over, unless it be engaged in the bend of the ham of the other leg, as we sometimes meet with it, for then we must begin by bringing down the foot of that leg first. As soon as we have brought one foot out of the uterus, we must introduce the hand again to search for the other, either by tracing the same course, &c.—HEATH's Translation of Baudelocque, vol. ii. page 208.

" On ne peut pas fixer d'une manière générale la direction que la main doit suivre dans la matrice pour atteindre les pieds. Comme elle varie suivant la situation de l'enfant, je ne puis indiquer le chemin le plus court et le plus facile pour parvenir jusqu'aux pieds que lorsque je traiterai de chaque position en particulier. Lorsque les pieds sont éloignés de l'orifice et pressés dans la matrice, il est necessaire de les entraîner tous les deux. Lorsque les eaux sont écoulées depuis long-temps, on arracherait plutôt le pied que d'amener l'enfant en tirant sur un seul: le plus souvent même on ne peut les extraire que l'un après l'autre. Dans les manœuvres que je vais décrire, je les supposerai toujours tellement serrés ou embarrassés l'un dans l'autre, que l'on ne peut pas les entrainer en même temps."—GARDIEN Traité complet d'Accouchemens, &c. tome ii. p. 443.

" Après avoir reconnu la partie qui se présente, on la refoule pour débarrasser le détroit supérieur, en ayant soin d'appliquer les doigts sur une surface aussi étendue que possible; puis on va à la recherche des membres, qui l'on saisit pour les ramener à l'orifice."—VELPEAU, Traité Elémentaire de l'Art des Accouchemens, &c., tome ii. p. 709.

" La plupart des accoucheurs ont conseillé et conseillent encore de saisir les pieds, et non pas une autre partie des membres; les pieds, enfin, sont la seule partie sur laquelle on recommande de tirer dans la version dont il s'agit. Cependant il est possible, il est même avantageuse, dans beaucoup de cas, de suivre le conseil donné d'abord par Burton, reproduit ensuite par M. Delpech, et tout récemment par M. Breen, c'est-à-dire de saisir les genoux ou les jarrets plutôt que les pieds."—Idem, p. 711.

" He should endeavour to pass the hand immediately to that part of the uterus where he expects to find the feet, and this must be determined by the presentation or situation of the child. When the operator has gained the feet, he should, 1st, grasp them firmly with the hand, but should always, in doing this, place a finger between them, to prevent injury by compressing them too strongly; 2d, when practicable, he always brings both feet along at the same time; 3d, though sometimes practicable, nay, easy occasionally, to deliver by one foot, it should never be done but from downright necessity, and this can occur but very rarely; 5th, should he be able to bring only one foot to the entrance of the vagina, let him secure it by a fillet, while he searches for the other."—Dr. DEWEES' Compendious System of Midwifery, p. 285.

The foregoing quotations prove that the operation of turning, as at present practised, consists in dilating the os externum, vagina, and os uteri, passing the hand into the uterus, searching for one foot, and then for the other, bringing down the feet to the os externum, and forthwith extracting the child. If the difficulty is very great in finding both feet, we are then allowed to content ourselves in bringing down one foot only.

The presentations of different parts of the child's body ascertained during labour, before the os uteri is dilated, are not all equally safe to the mother or the child. Presentations of the vertex are accepted as natural, because they are the most frequent, the most safe, and are capable of being accomplished by the unaided powers of nature. The different presentations of the head constitute varieties, but are nevertheless more safe both to the mother and child than any other. The presentation which ranks the next in point of safety is the breech, after which are placed presentations of the lower extremities. In labours where the trunk, or superior extremities of the child present, the process cannot be completed by the natural powers, except in those rare cases where spontaneous evolution takes place. If the mother were left to her own powers unaided, she would inevitably perish, and also her offspring.

In these cases, therefore, manual assistance becomes necessary. The practice is evidently that of changing the position of the child, so that the natural powers of the mother may accomplish its expulsion, or that it may be delivered by the accoucheur. The means most desirable to adopt in such cases, would be, if possible, to change the presentation to that of the head, this being the safest and most natural. This practice was inculcated and attempted by the older writers, but cannot be put in execution, from the inability to seize a body of such magnitude as the child's head, so as to bring it to lie over the os uteri. As the breech is the presentation next in point of safety, we might on this account be induced to seize this part to effect a change of position, but the same objections exactly apply to this as to the attempt at bringing down the head. We are therefore reduced to the necessity of adopting the present practice of bringing down the feet. A superficial view of these cases might lead us to the conclusion, that if it were possible to effect a breech presentation, little comparative advantage would be obtained over bringing down the feet. But the results of practice prove, what might be inferred by reasoning, that the child's life is much more frequently preserved in those cases in which it presents the breech, than where the feet come down first. The measurement of the child round the hips and thighs, when the latter are turned up towards the belly, as in breech cases, approaches nearly to the circumference of that portion of the head which lies parallel with the pelvic cavity in natural labour. The dilatation of the os uteri and os externum is effected so completely by the passage of the breech, that very little resistance is offered to the expulsion of the head, or a dexterous extraction of it, if the umbilical chord should shew signs of compression hazarding the life of the child. But this is not the case in presentations of the lower extremities; the child in this case descends in a wedge-like form, constantly presenting an increase of measurement in the successive parts of its body; which parts, in comparison with the head are rather more yielding. At the time the head, which presents the greatest circumference, enters the pelvis, compression of the funis takes place, and if the cervix and os uteri are not sufficiently dilatable, which is most generally the case, especially in first labours, the child will be lost. The attempt to bring the child rapidly through the pelvis has a tendency to increase the opposition by inducing a spasmodic action of the cervix, which, coupled with the inadequate dilatability of the os uteri, becomes *pro tempore* an insurmountable barrier to the extraction. Is there, then, no practice which would enable us to bring down a part, approximating in its measurements to that of the breech presentation which we have already stated to be so safe to the child, but which cannot be effected? There is, and this practice consists in never bringing down more than one foot in the manual operation of turning the child.

The propriety of this practice may be shewn by considering the greater safety of breech presentations over those of the feet, and comparing the measurements of the breech with the thighs flexed upon the pelvis with that of the head. If, as above stated, we are not able to effect a breech presentation by the hand in utero, that operation which produces a presentation possessing a measurement nearest to it is the one we ought to prefer, and this consists in bringing down one foot only, leaving the other, which would become turned up towards the abdomen of the child, thus producing a presentation compounded of the breech and one lower extremity. The circumference of the hips in this position of the parts varies very little from that of pure breech cases, and must consequently be attended by nearly all the advantages of such a case. In considering this subject, it will be desirable to attend carefully to the subjoined measurements, which have been accurately obtained from children born at the full period of gestation. The circumference of that portion of the head which presents in labour is from twelve to thirteen and a half inches. Ditto of the breech with the thighs turned upwards to the belly, as in breech presentations, from twelve to thirteen and a half inches. Ditto with one thigh turned up towards the abdomen, the other extended, from eleven to twelve and a half inches. Ditto of the hips, the legs extended as in feet presentations, from ten to eleven and a half inches.

These measurements prove several points. 1st. That breech cases differ very little in their power to dilate the passages of the mother as extensively as the head, and therefore that whenever the breech passes, we may expect the head to pass with moderate ease, either by the natural efforts or by judicious artificial aid. They also show, 2dly, That when one thigh is turned up towards the abdomen of the child, that the measurement is very little less than that of the presenting part in pure breech cases, and therefore that we may expect the head to pass with nearly as much ease as in those cases.

3dly. That where both feet are down, the measurement of the hips differs so much from that of the head as to be insufficient to accomplish that dilatation of the soft parts which will allow the head to pass, or be extracted with sufficient celerity to save the child, when its life is threatened by the compression of the funis.

The foregoing observations authorise the conclusion, that in undertaking this operation we should make it a positive and invariable rule never to bring down more than one foot or one knee.

Whenever any such circumstances occur during labour, as large hemorrhages, preternatural presentations, &c. for the relief of which we adopt the practice of turning, the uterus will be found in one or other of the following conditions: either fully distended, the liquor amnii not having escaped; or more or less contracted upon the body of the child, the liquor amnii having been discharged. In the first class of cases the operation is effected in a short space of time with great ease to the practitioner, and with comparative safety to the mother and the child. In the second class of cases, where the liquor amnii has escaped, and the contraction of the uterus is more or less powerful and closely embracing the child, the difficulties and dangers of turning manifest themselves. In these cases we are recommended to bring down both feet; but if we find great difficulty in doing so, to content ourselves by bringing down one foot only.

The interrogatory is naturally suggested by this last sentence, Why? The answer to which is, because it is more easy and more safe to both mother and child to do so. In rules given by authors for the management of these cases, we are restricted to the operation most complex to the practitioner, most difficult to the mother, and most dangerous to the child, for the relief of that class which are of easy accomplishment; whilst we have the option only of adopting the most easy plan in the most serious and difficult cases.

In all cases, when the liquor amnii has been discharged, it is our special duty to bring down one foot only. If but a short time has elapsed since this occurrence, the case will still maintain a favorable aspect, the presenting part will not be wedged in the superior aperture of the pelvis, the uterus will not be powerfully contracted upon the body of the child, and the operation of turning may be accomplished with moderate facility and additional safety by bringing down only one foot.

But when the liquor amnii has been long evacuated, the case assumes a different character; the presenting part is wedged firmly in the superior aperture of the pelvis, the uterus is powerfully embracing the body of the child, the os uteri is hard, tumefied, and indisposed to dilate. The hand of the operator is impeded by the arm of the child at the os externum, and all the parts exposed to the mechanical influence of pressure, have sustained injury from the long-continued but ineffective action. In such cases we should never contemplate bringing down two feet, not abandoning this plan, because we cannot find, or cannot secure the second foot, but should make the principle of our operation the bringing down one foot only; and where the contraction of the uterus upon the child's body is so strong as to create difficulty, we should not search for the foot, provided we can secure and bring down one knee; for as the knee is brought down, the foot will descend and come within reach, so that it may be seized and brought through the os uteri with facility. In such a condition of the organs, it is evident that every rule which has a tendency to lessen irritation and soothe and subdue the morbid sympathies, abundantly excited by the pressure of the child, and the manual movements of the operator, is of the most vital importance to the female. Great caution should be observed in the mode of introducing the hand not only through the os uteri but along the vaginal canal; it is also of great consequence to attend to the length of the period during which the hand remains introduced. In accordance with the principles so frequently urged, it will be manifest that the evolution of the child, and the easy recession of the presentation, will be better accomplished by not accumulating too great a bulk upon this part at the os uteri. These extreme cases of difficulty in turning are most commonly met with in preternatural presentations neglected in the early stages, and often attendant upon the practice of females. I have never yet met with a case where the liquor amnii has been evacuated for more than twenty-four hours, where the difficulty of turning was not increased by a strong circular band of uterine muscular fibres, which embraced closely and firmly the lower part of the chest and the abdomen of the child, leaving the breech and inferior extremities in a kind of chamber at the fundus; the head, shoulder, and inferior extremities lying beneath, and the presenting part protruding through the os uteri, which is partially dilated. The practice before stated I have the more confidence in recommending, as it corresponds with the views of my valued relative Mr. Wood, whose opportunities of observation in this department have been most extensive, and such as rarely fall within the sphere of one individual.

I have purposely omitted entering into a detail of the several preliminary measures calculated to facilitate the operation; the rules regulating the choice of the hand; the mode of proceeding to turn; the several steps in the extraction of the child, and also the consideration of the practicability of turning in all cases. These may perhaps form the subject of a future communicatlon.—*Edinburgh Med. and Surg. Journal.*

---

### MISCELLANEOUS.

*Test of the Choleric Diathesis.* We know not with whom the suggestion originated, but, for the last ten days, we find that litmus paper has been in considerable demand as a test of the choleric diathesis. It is said that the predisposition to become affected with cholera is evidenced by a morbid developement of acid in the system generally, which may be detected in the saliva by placing a piece of litmus paper beneath the tongue; when, if the blue tint remain unaltered, the person may be esteemed comparatively safe; but if it assume a reddish hue, then let him beware.

---

*Zoology.* The smell of burning assafœtida has a remarkable effect upon wolves. If a fire be made in the woods, and a portion of this drug be thrown into it, so as to saturate the atmosphere with the odour, the wolves, if any are within reach of the scent, immediately assemble around, howling in the most mournful manner; and such is the remarkable fascination under which they seem to labour, that they will suffer themselves to be shot down rather than quit the spot.—*Featherstonehaugh's Journal.*

### INTELLIGENCE.

